# Identification of the immune checkpoint signature of multiple myeloma using mass cytometry‐based single‐cell analysis

**DOI:** 10.1002/cti2.1132

**Published:** 2020-04-29

**Authors:** Jinheng Wang, Yongjiang Zheng, Chenggong Tu, Hui Zhang, Karin Vanderkerken, Eline Menu, Jinbao Liu

**Affiliations:** ^1^ Affiliated Cancer Hospital & Institute of Guangzhou Medical University Guangzhou Municipal and Guangdong Provincial Key Laboratory of Protein Modification and Degradation State Key Laboratory of Respiratory Disease School of Basic Medical Sciences Guangzhou Medical University Guangzhou China; ^2^ Department of Hematology The Third Affiliated Hospital of Sun Yat‐Sen University Guangzhou China; ^3^ Department of Hematology and Immunology Myeloma Center Brussels Vrije Universiteit Brussel Brussels Belgium

**Keywords:** immune checkpoint, immunotherapy, mass cytometry, multiple myeloma, single‐cell analysis

## Abstract

**Objectives:**

New targets or strategies are needed to increase the success of immune checkpoint‐based immunotherapy for multiple myeloma (MM). However, immune checkpoint signals in MM microenvironment have not been fully elucidated. Here, we aimed to have a broad overview of the different immune subsets and their immune checkpoint status, within the MM microenvironment, and to provide novel immunotherapeutic targets to treat MM patients.

**Methods:**

We performed immune checkpoint profiling of bone marrow (BM) samples from MM patients and healthy controls using mass cytometry. With high‐dimensional single‐cell analysis of 30 immune proteins containing 10 pairs of immune checkpoint axes in 0.55 million of BM cells, an immune landscape of MM was mapped.

**Results:**

We identified an abnormality of immune cell composition by demonstrating a significant increase in activated CD4 T, CD8 T, CD8^+^ natural killer T‐like and NK cells in MM BM. Our data suggest a correlation between MM cells and immune checkpoint phenotypes and expand the view of MM immune signatures. Specifically, several critical immune checkpoints, such as programmed cell death 1 (PD‐1)/PD ligand 2, galectin‐9/T‐cell immunoglobulin mucin‐3, and inducible T‐cell costimulator (ICOS)/ICOS ligand, on both MM and immune effector cells and a number of activated PD‐1^+^ CD8 T cells lacking CD28 were distinguished in MM patients.

**Conclusion:**

A clear interaction between MM cells and the surrounding immune cells was established, leading to immune checkpoint dysregulation. The analysis of the immune landscape enhances our understanding of the MM immunological milieu and proposes novel targets for improving immune checkpoint blockade‐based MM immunotherapy.

## Graphical abstract

In this study, we performed immune checkpoint profiling of bone marrow (BM) samples from multiple myeloma (MM) patients and healthy controls using mass cytometry. Our data suggest a correlation between MM cells and immune checkpoint phenotypes and expand the view of MM immune signatures. Specifically, several critical immune checkpoints, such as PD‐1/PD‐L2, galectin‐9/T‐cell immunoglobulin mucin‐3 and ICOS/ICOSL, on both MM and immune effector cells and a number of activated PD‐1^+^ CD8 T cells lacking CD28 were distinguished in MM patients, and they serve as novel targets for developing more potent and efficacious checkpoint blockade‐based MM immunotherapeutic strategies.

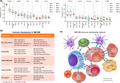

## Introduction

Multiple myeloma (MM) is a cancer of clonal plasma cells preferentially localised in the bone marrow (BM). The proliferation of MM cells, together with an MM cell‐changed BM microenvironment, suppresses local and systemic immunity, eventually leading to an escape from immune surveillance.^1^ Mechanisms involved in MM‐induced immunosuppression include dysfunction of T and natural killer (NK) cells,^2^ disruption of antigen presentation processes,^3^ activation of immunosuppressive cells,^3^, ^4^ upregulation of inhibitory immune checkpoints^5^, ^6^ and release of immunosuppressive mediators.^7^ Comprehensively uncovering the immune status in the BM microenvironment of MM patients will largely facilitate the understanding of the ongoing process of immunosuppression in MM progression and therefore promote the development of novel immunotherapeutic strategies.

Immunotherapy that involves stimulating and provoking a patients' own immune system against cancer has proven to be very encouraging as dramatic and durable anticancer responses are well documented in many cancer types.^8^, ^9^ Blocking inhibitory immune checkpoints on immune effector cells results in the reactivation of anticancer immunity.^10^ Immune checkpoints contain a series of costimulatory and coinhibitory receptors or ligands expressed on T, NK or antigen‐presenting cells and mainly function as switches of immune activation or suppression.^11^ Under normal physiological conditions, immune checkpoints maintain self‐tolerance and immune homeostasis, whereas malignant cells take advantage of these molecules to achieve immune evasion.^12^ The most prominent immune checkpoint blocking strategies, such as targeting cytotoxic T lymphocyte‐associated protein 4 (CTLA‐4) and blocking the interaction between programmed cell death 1 (PD‐1) and PD ligand 1 (PD‐L1), are able to enlist and strengthen the immune system to attack cancer cells and have achieved clinical success in several cancer types, even in metastatic and chemoresistant cancer.^13^, ^14^ However, these immunotherapies are unable to control malignancy in a significant proportion of patients, largely because of the fact that inhibitory signals inducing the exhaustion and dysfunction of anticancer immune cells are not fully and sustainably blocked.^10^, ^15^ Indeed, as reported by a phase 1b clinical study, PD‐1/PD‐L1 axis‐based immune checkpoint blockade failed to control MM progression,^16^, ^17^ suggesting that this checkpoint may not be the major mediator of failing anti‐MM immunity. Besides PD‐1 and CTLA‐4, many other immune checkpoints have been discovered and are used for improved immune checkpoint‐based immunotherapy.^18^ However, immune checkpoint signals in the MM microenvironment have not been fully elucidated. The analysis of immune checkpoints will help us to better understand the mechanism of immune evasion of MM cells and would allow the development of potent strategies, focused on the checkpoint signals that are actually used by MM cells to evade the immune system.

The most commonly used technique for immune phenotyping, flow cytometry, suffers from the limited detection channels (generally < 15) and cumbersome compensation because of spectral overlap, making it difficult to simultaneously detect all immune checkpoint phenotypes. As a cutting‐edge single‐cell technology, current mass cytometry merging mass spectrometry with flow cytometry permits up to 50 metal isotope tags to be measured simultaneously on a single cell with minimal/no compensation.^19^, ^20^ Such high multiparametric detection provides an unprecedented opportunity for deep phenotyping of the tumor immune microenvironment at the single‐cell level. For now, this powerful innovation has offered insights into the heterogeneity and complexity of biology and has been used to understand the complex processes in cellular development,^21^ differentiation^22^ and tumor immunology,^23^, ^24^, ^25^ and to explore the potential immunotherapeutic targets.^23^

In this study, 0.55 million BM cells from 10 MM patients and five healthy donors (HD) were analysed using mass cytometry to elucidate the phenotypic diversity and immune checkpoint signature in MM BM ecosystems. Our data reveal vast phenotypic heterogeneity among both malignant and immune cells, identify an abnormality of immune cell composition and suggest links between MM cells and immune checkpoint phenotypes. Through in‐depth analyses of 10 pairs of immune checkpoint axes in 12 identified immune cell types at the single‐cell level, a picture of the immune checkpoint interaction network that exists in the MM BM microenvironment of these patients was established. Several critical immune checkpoints were identified in the MM BM and may serve as novel targets for developing more potent and efficacious checkpoint blockade‐based MM immunotherapeutic strategies.

## Results

### In‐depth immune checkpoint phenotyping of MM cells using mass cytometry

**Figure 1:**
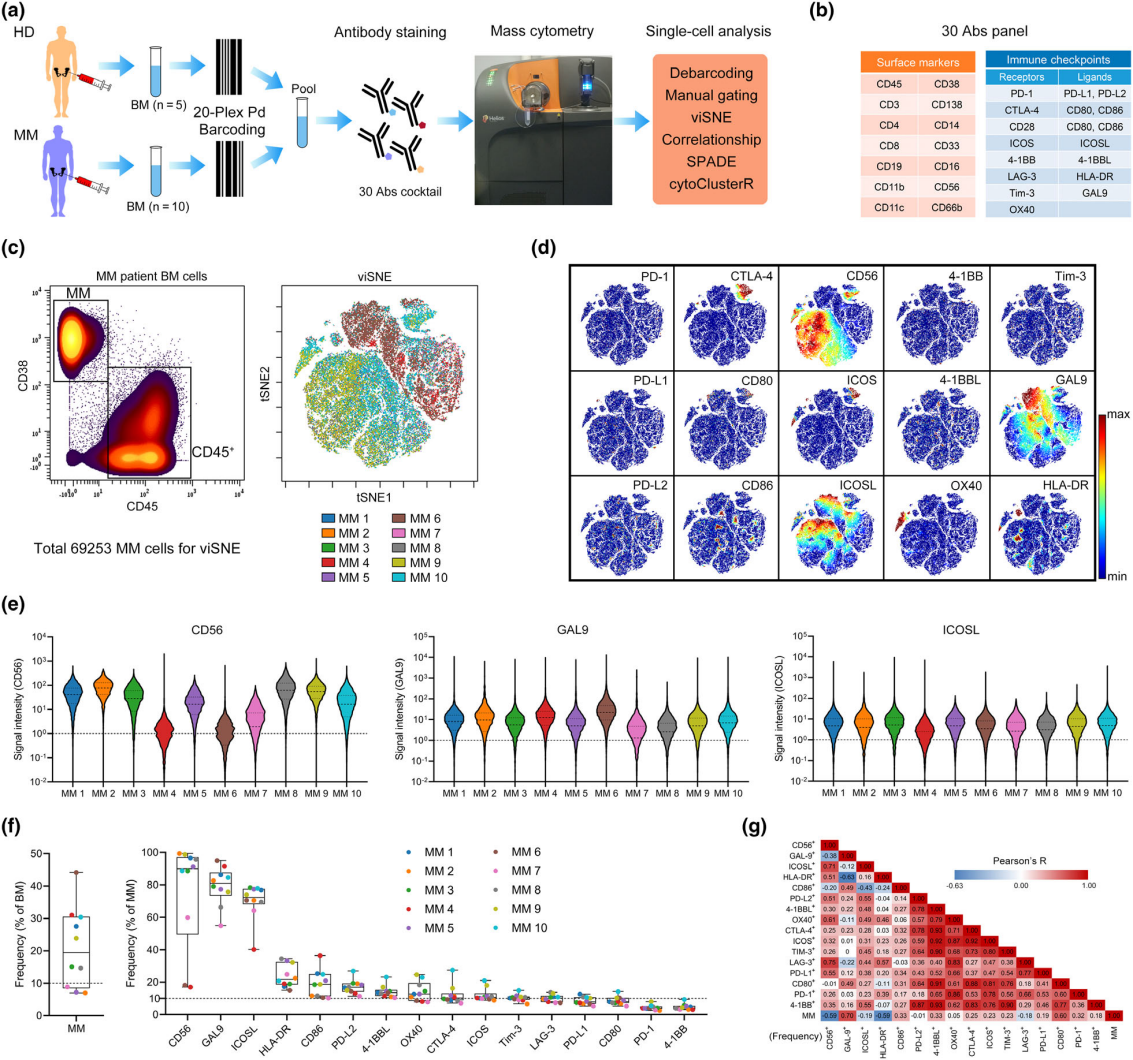
Characterisation of immune checkpoints of MM cells. **(a)** The experimental workflow used in this study. **(b)** Markers used to define cell populations and immune checkpoint phenotypes. **(c)** Gating of MM and CD45^+^ cells (left panel). viSNE map showing 69 253 MM cells from the BM of MM (*n* = 10) patients coloured by individual. **(d)** Cells coloured by normalised expression of indicated immune checkpoint markers on the viSNE map. **(e)** A violin plot showing the signal intensity of CD56, GAL9 and ICOSL in MM cells of individual patients. **(f)** Dot plots showing the frequency of MM cells among BM cells (left panel) and indicated markers' positive cells among MM cells for each MM BM sample (right panel). Dots are coloured by individual. **(g)** A heatmap showing the Pearson correlation coefficients for relationships between the frequencies of indicated cell populations. Abs, antibodies; BM, bone marrow; HD, healthy donor; MM, multiple myeloma. MM, *n* = 10.

To map the immune checkpoint signatures in the BM microenvironment of MM patients, we implemented a clinical high‐dimensional single‐cell profiling study of freshly collected BM from newly diagnosed and untreated MM patients using mass cytometry. Ten MM BM samples and five healthy BM samples were included for a large‐scale mass cytometry analysis (Figure 1a). We stained prebarcoded BM cells with 30 antibodies to simultaneously determine the expression of 30 markers used to define cell populations and immune checkpoint phenotypes at the single‐cell level (Figure 1b). As the loss of CD138 caused by the cold storage and processing frequently occurs,^26^, ^27^ cells with a CD38^++^CD45^−/dim^ phenotype were defined as malignant MM cells (Figure 1c). To comprehensively view the immune checkpoint profile of MM cells from all patients, we generated a single‐cell viSNE map to visualise high‐dimensional data in two dimensions.^28^ This analysis demonstrated a clear heterogeneity of MM cells among patients (Figure 1c). On the viSNE map, clear expression of multiple immunoregulatory proteins, including CTLA‐4, CD56, inducible T‐cell costimulator (ICOS), galectin‐9 (GAL9), CD86, ICOS ligand (ICOSL), OX40 and HLA‐DR, was observed in different MM cell clusters (Figure 1d). Large proportion of CD56^+^ MM cells were detected in 8 of 10 patients, and GAL9 and ICOSL expressions were widely found in MM cells from all patients (Figure 1e), whereas high PD‐L1 or PD‐L2 expressions were only observed in few MM cells (Supplementary figure 1a). These 10 BM samples with 7–41% MM cells displayed diverse phenotypes in the expression of immune checkpoint proteins. Important immune checkpoint ligands, including GAL9, ICOSL, HLA‐DR, CD86, PD‐L2, and 4‐1BBL, were expressed by more than 10% of MM cells in average (Figure 1f and Supplementary figure 1b). We next performed correlation analyses to systematically quantify the underlying relationships between overall MM burden and MM cells with different immune checkpoint phenotypes. Multiple robust either positive or negative relationships were identified (Figure 1g). Among the positive relationships, GAL9 expression was most strongly related to MM burden. Also, the expression of different immune checkpoint ligands correlated significantly with each other, such as PD‐L2 expression, which correlated with 4‐1BBL and CD56 expressions with ICOSL (Supplementary figure 1c).

### Immune cell signature in MM BM microenvironment

**Figure 2:**
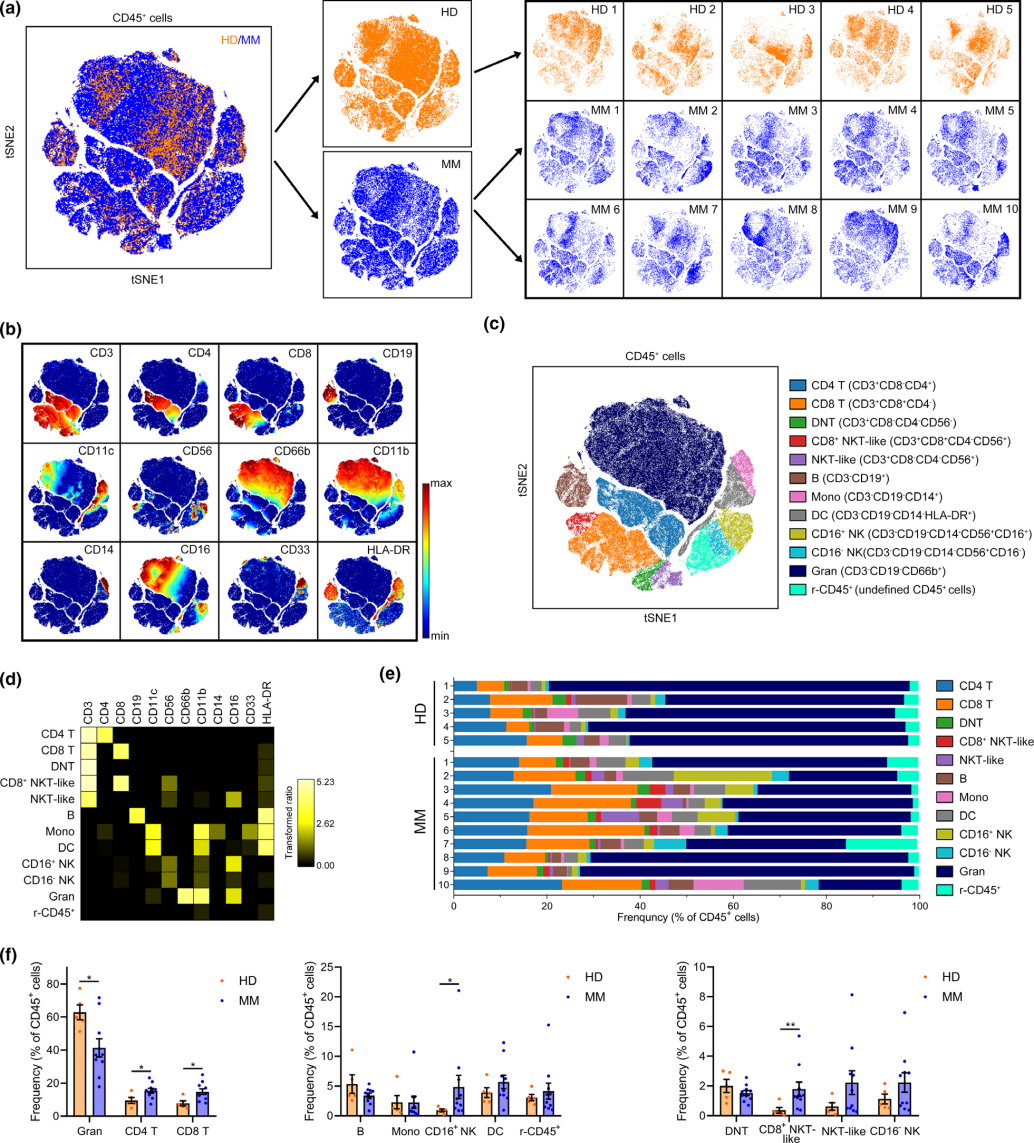
Immune cell population changes in the BM of MM patients. **(a)** A viSNE map displaying gated CD45^+^ BM cells of five HD and 10 MM patients coloured by groups. **(b)** A viSNE map coloured by the normalised expression of indicated markers. **(c)** A viSNE map coloured by 12 main cell populations after clustering. **(d)** A heatmap showing the normalised median expression of 12 indicated markers in 12 cell populations. **(e)** Frequencies of 12 cell populations in CD45^+^ cells for each BM sample. Cell types are indicated by colour. **(f)** Bar plots showing the frequencies of indicated populations in BM CD45^+^ cells of HD and MM patients. HD, *n* = 5; MM, *n* = 10. DC, dendritic cells; DNT, double‐negative T; Gran, granulocytes; Mono, monocytes; NK, natural killer; r‐CD45^+^, the rest of CD45^+^. *P < 0.05 and **P < 0.01.

Next, we used viSNE to visualise the distribution of the immune cells in the HD and MM BM samples (equal cell number from each individual) and demonstrated a large heterogeneity among MM patients and healthy controls (Figure 2a). According to the standardised immuno‐phenotyping for human immunology^29^ and the expression of 15 surface markers in HD and MM BM CD45^+^ cells displayed on the viSNE map (Figure 2b), 12 major immune cell populations were gated on the map (Figure 2c). Natural killer T (NKT) cells are identified with a CD3^+^CD56^+^ phenotype in many studies.^30^, ^31^, ^32^ However, only a small proportion of CD3^+^CD56^+^ are CD1d‐restricted, which is a unique feature of invariant NKT (iNKT) cells. Thus, this population is frequently referred to as ‘NKT‐like’.^32^ Here, we gated two CD3^+^CD56^+^ cell subsets, namely NKT‐like and CD8^+^ NKT‐like cells, after excluding CD4, CD8 and double‐negative (DN) T cells from all CD3^+^ cells. As shown by heatmap, the expression of surface markers in each population was identical to the phenotype of indicated immune lineages (Figure 2d). After gating on viSNE map, the immune lineages in individual samples were analysed (Figure 2e), which revealed a heterogeneity across HD or MM patients. Although wide variation existed in the frequencies of each immune cell type in different individuals, several significant changes between HD and MM patients were detected. In the BM of MM patients, the proportion of CD4 T, CD8 T, CD16^+^ NK and CD8^+^ NKT‐like cells in CD45^+^ immune cells was significantly increased along with the significant decrease in granulocytes, as compared to those in HD BM cells (Figure 2f). The average percentage of CD8 T cells increased from 7.77% in HD to 14.82% in MM and that of CD4 T cells rose from 9.49% to 15.36%. Importantly, CD8^+^ NKT‐like cells only accounted for 0.92% of HD BM immune cells in average, whereas it increased to 4.86% in MM patients (Figure 2f). iNKT cells have been shown to be associated with MM and are important for antitumor immunity.^33^ We also examined the proportion of iNKT cells with the T‐cell receptor Va24Ja18 antibody. We found that they constitute a minor fraction of BM T cells and there is no significant difference in their percentages between HD and MM patients (Supplementary figure 2a). Moreover, the MM burden was positively correlated with the frequency of CD8 T cells in MM patients and negatively correlated with the frequency of CD16^+^ NK cells with a trend close to significance (Supplementary figure 2b).

### The immune checkpoint landscape of MM BM T cells

**Figure 3:**
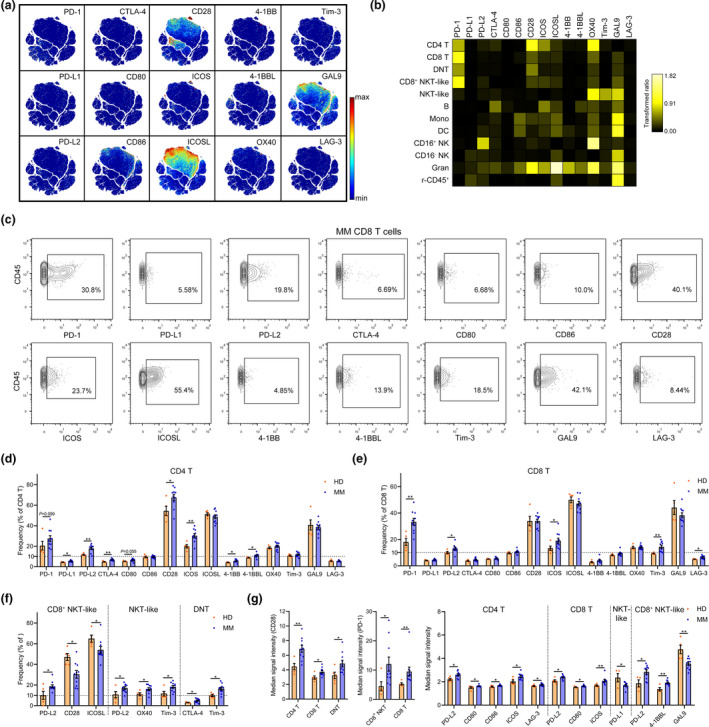
Immune checkpoint changes in MM BM T cells. **(a)** A viSNE map coloured by the normalised expression of 15 immune checkpoint markers. **(b)** Heatmaps showing the normalised mean expression of 15 immune checkpoint markers in all cell populations (normalised to the column's minimum). **(c)** Contour plots showing the gating strategy and the expression of indicated checkpoint molecules in CD8 T cells of one representative MM patient. **(d, e)** Bar plots showing the frequencies of indicated markers' positive cells in BM **(d),** CD4 and **(e)** CD8 T cells of HD and MM patients. **(f)** Bar plots showing the significantly changed frequencies of indicated markers' positive cells in CD8^+^ NKT‐like, NKT‐like and DNT cells of HD and MM patients. **(g)** Bar plots showing the significantly changed median signal intensity of indicated markers in corresponding positive T‐cell subsets of HD and MM patients. HD, *n* = 5; MM, *n* = 10. *P < 0.05 and **P < 0.01.

To characterise the immune checkpoint phenotype in MM BM immune cells, we assessed the expression of all detected immune checkpoint proteins in CD45^+^ cells on the viSNE map. ICOSL, CD28, CD86 and GAL9 expressions were clearly observed in several cell subsets (Figure 3a). In contrast, no clear accumulative expression of the other immune checkpoints appeared on the viSNE map. However, the normalised mean expression of these proteins was distinct among the 12 gated immune cell populations clustered from viSNE map (Figure 3b), suggesting the presence of heterogeneous subgroups with high immune checkpoint expression in these populations. Thus, we first compared the frequencies of immune checkpoint‐positive cells in all cell populations of HD BM with those of MM patients (Figure 3c and Supplementary figure 3). In BM CD4 T‐cell subsets, the proportions of PD‐L1^+^, PD‐L2^+^, CTLA‐4^+^, 4‐1BB^+^ and 4‐1BBL^+^ cells were consistently < 20%, but were significantly higher in MM patients than those in HD. The percentages of CD28^+^ and ICOS^+^ CD4 T cells were also significantly higher in MM patients than in HD (Figure 3d and Supplementary figure 4a). Moreover, PD‐1^+^, PD‐L2^+^, ICOS^+^, T‐cell immunoglobulin mucin‐3 (Tim‐3)^+^ and lymphocyte activating 3 (LAG‐3)^+^ CD8 T cells were significantly increased in the BM of MM patients (Figure 3e and Supplementary figure 4b). Additionally, several immune checkpoints were also significantly increased in other T‐cell types, such as the number of PD‐L2^+^ cells in CD8^+^ NKT‐like cells; PD‐L2^+^, OX40^+^ and Tim‐3^+^ cells in NKT‐like cells; and CTLA‐4^+^ and the number of Tim‐3^+^ cells in DNT cells. By contrast, some decreases in the number of immune checkpoint‐positive cells were observed as well, such as CD28^+^ and ICOSL^+^ cells in CD8^+^ NKT‐like cells (Figure 3f and Supplementary figure 4c–e). We also compared the intensity of the expression of these checkpoints in the corresponding positive cells. The expression of CD28 was significantly stronger in CD28^+^ CD4 T, CD8 T and DNT cells of MM patients. In PD‐1^+^ CD8^+^ NKT‐like and CD8 T cells, the PD‐1 expression was also significantly increased in MM patients. Many significant changes in the expression of immune checkpoints in CD4 T, CD8 T, NKT‐like or CD8^+^ NKT‐like cells were discovered (Figure 3g).

### The immune checkpoint atlas of MM BM non‐T cells

**Figure 4:**
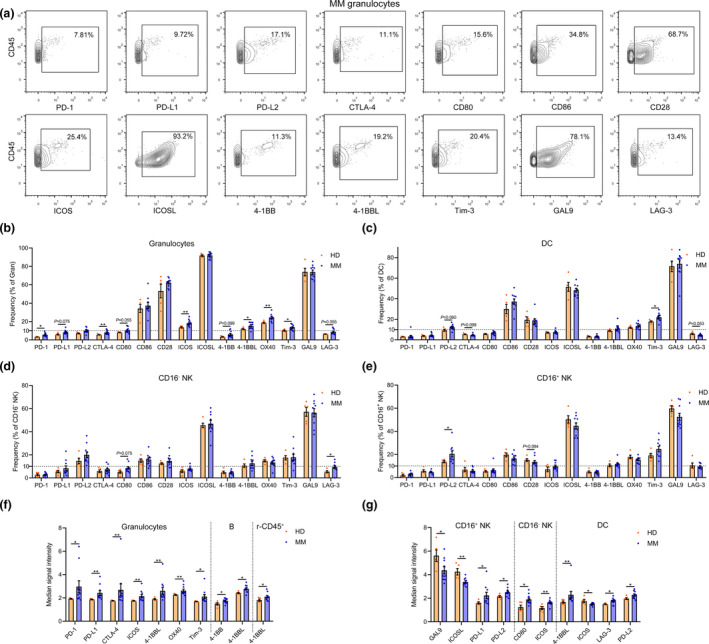
Immune checkpoint changes in MM BM non‐T immune cells. **(a)** Contour plots showing the gating strategy and the expression of indicated checkpoint molecules in granulocytes of one representative MM patient. **(b–e)** Bar plots showing the frequencies of indicated markers' positive cells in BM **(b)** granulocytes, **(c)** DC, **(d)** CD16^−^ NK and **(e)** CD16^+^ NK cells of HD and MM patients. **(f, g)** Bar plots showing the significantly changed median signal intensity of indicated markers in corresponding positive **(f)** granulocytes, B and r‐CD45^+^, and **(g)** DC, CD16^+^ and CD16^−^ NK cells of HD and MM patients. HD, *n* = 5; MM, *n* = 10. *P < 0.05 and **P < 0.01.

The frequencies of PD‐1^+^, PD‐L2^+^, CTLA‐4^+^, ICOS^+^, 4‐1BBL^+^, OX40^+^ and Tim‐3^+^ cells in granulocytes were significantly increased in MM patients, although most of them were < 10% (Figure 4a and b). Granulocytes accounted for the major provider of ICOSL as more than 85% of them express ICOSL in both HD and MM patients. The frequencies of PD‐1^+^ and 4‐1BB^+^ cells in undefined (the rest of) CD45^+^, Tim‐3^+^ cells in DC, LAG‐3^+^ cells in CD16^−^ NK cells, and PD‐L2^+^ cells in CD16^+^ NK cells were also significantly increased in MM patients (Figure 4c–e and Supplementary figure 5a–c). Although significant differences in the percentages of immune checkpoint‐positive cells were not detected in many cell types, the intensity of their expression in several immune cell populations was significantly altered in MM patients (Figure 4f and g).

### Activation signature of T and NK cells in MM BM microenvironment

**Figure 5:**
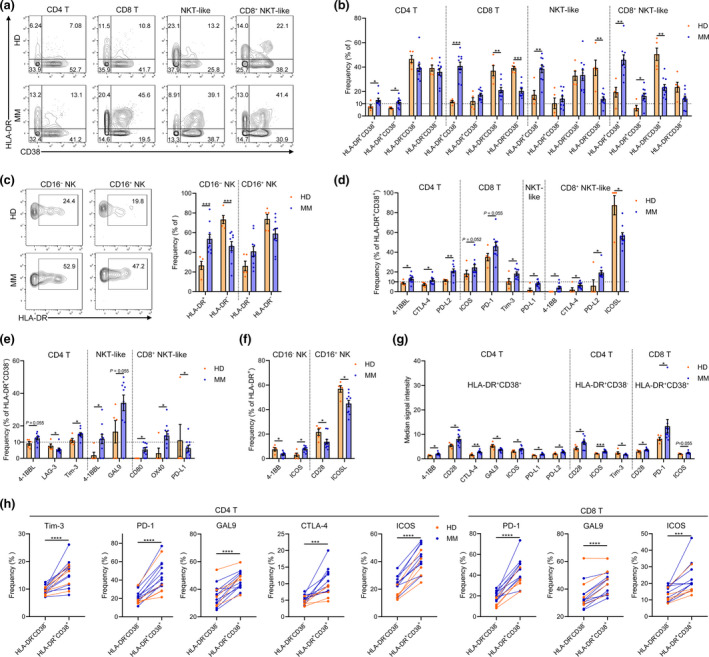
Changes in T‐cell activation status in the BM of MM patients. **(a)** Contour plots showing the expression of CD38 and HLA‐DR in 4 T‐cell subsets of one representative HD or MM patient. **(b)** Bar plots showing the frequencies of indicated cell clusters in BM T‐cell subsets of HD and MM patients. **(c)** Contour plots showing the expression of HLA‐DR in NK cell subsets of one representative HD or MM patient (left panel). Bar plots showing the frequencies of indicated clusters in BM NK cell subsets of HD and MM patients (right panel). **(d)** Bar plots showing the significantly changed frequencies of indicated markers' positive cells in HLA‐DR^+^CD38^+^ T‐cell subsets of HD and MM patients. **(e)** Bar plots showing the significantly changed frequencies of indicated markers' positive cells in HLA‐DR^+^CD38^−^ T‐cell subsets of HD and MM patients. **(f)** Bar plots showing the significantly changed frequencies of indicated markers' positive cells in HLA‐DR^+^ NK cell subsets of HD and MM patients. **(g)** Bar plots showing the significantly changed median signal intensity of indicated markers in corresponding positive HLA‐DR^+^CD38^+^ CD4 T, HLA‐DR^+^CD38^−^ CD4 T and HLA‐DR^+^CD38^+^ CD8 T cells of HD and MM patients. **(h)** Dot plots showing the significantly changed frequencies of the indicated markers' positive cells in HLA‐DR^−^CD38^−^ and HLA‐DR^+^CD38^+^ T‐cell subsets of the individual. HD, *n* = 5; MM, *n* = 10. *P < 0.05, **P < 0.01, ***P < 0.001 and ****P < 0.0001.

CD8 T and NK cells are major contributors to anticancer immunity and the main targets to be reinvigorated by immune checkpoint blockade‐based immunotherapy. HLA‐DR appears at the late stages of activated T and NK cells and has been widely used as an activation marker.^34^, ^35^, ^36^ CD38 and HLA‐DR are also primarily regarded as biomarkers for identifying activated T cells.^29^, ^37^ Here, the activation status of T‐ and NK cell subsets was systematically quantified using these markers. In the MM BM cells, significant increase in activated (HLA‐DR^+^CD38^+^) cells was found in CD4 T, CD8 T, NKT‐like and CD8^+^ NKT‐like cells (Figure 5a). Specifically, the average frequency of activated cells in CD8 T cells was dramatically elevated from 11.66% in HD to 40.94% in MM patients (Figure 5b). Similarly, activated NK cells in the BM were also increased in MM patients (Figure 5c). A number of strong positive or negative correlations were revealed between the frequencies of activated T‐ or NK cell subsets and indicated immune checkpoint protein‐expressing MM cells in all patients (Supplementary figure 6a). In activated (HLA‐DR^+^) T‐ or NK cell subsets, several changes in immune checkpoint phenotype appeared in MM patients compared with those in HD (Figure 5d–f), changes were also found in inactivated (HLA‐DR^−^) T cells (Supplementary figure 6b and c). In addition, the expression of important immune checkpoints, including PD‐1, CD28 and ICOS, was changed in activated (HLA‐DR^+^) CD4 and CD8 T cells (Figure 5g). Coexpression of CD39 and CD103 has been used to identify the tumor‐specific CD8^+^ T cells in human tumors.^38^, ^39^ Here, we introduced these two markers to examine whether increased CD8 T cells in the BM are specific against MM cells. However, above 90% of CD8 T or activated CD8 T cells are CD39^–^ and CD103^–^negative (Supplementary figure 6d), suggesting that bystander T cells instead of tumor‐specific CD8 T cells are abundant in MM BM. To identify the immune checkpoint phenotypes in activated cells, we compared the frequencies of the immune marker‐expressing cells in inactivated with activated T or NK cells. Activated CD4 T cells expressed more Tim‐3, PD‐1, GAL9, CTLA‐4, ICOS and 4‐1BB than inactivated cells in both HD and MM patients. More activated CD8 T cells expressed PD‐1, GAL9, ICOS, CTLA‐4 and Tim‐3 (Figure 5h). Moreover, compared with inactivated cells, more activated NKT‐like cells expressed CTLA‐4; more activated CD16^−^ NK cells expressed CTLA‐4, Tim‐3 and GAL9; and more activated CD16^+^ NK cells expressed 4‐1BB (Supplementary figure 6e).

### In‐depth and systematic analyses of the immune checkpoint profile of MM BM T cells

**Figure 6:**
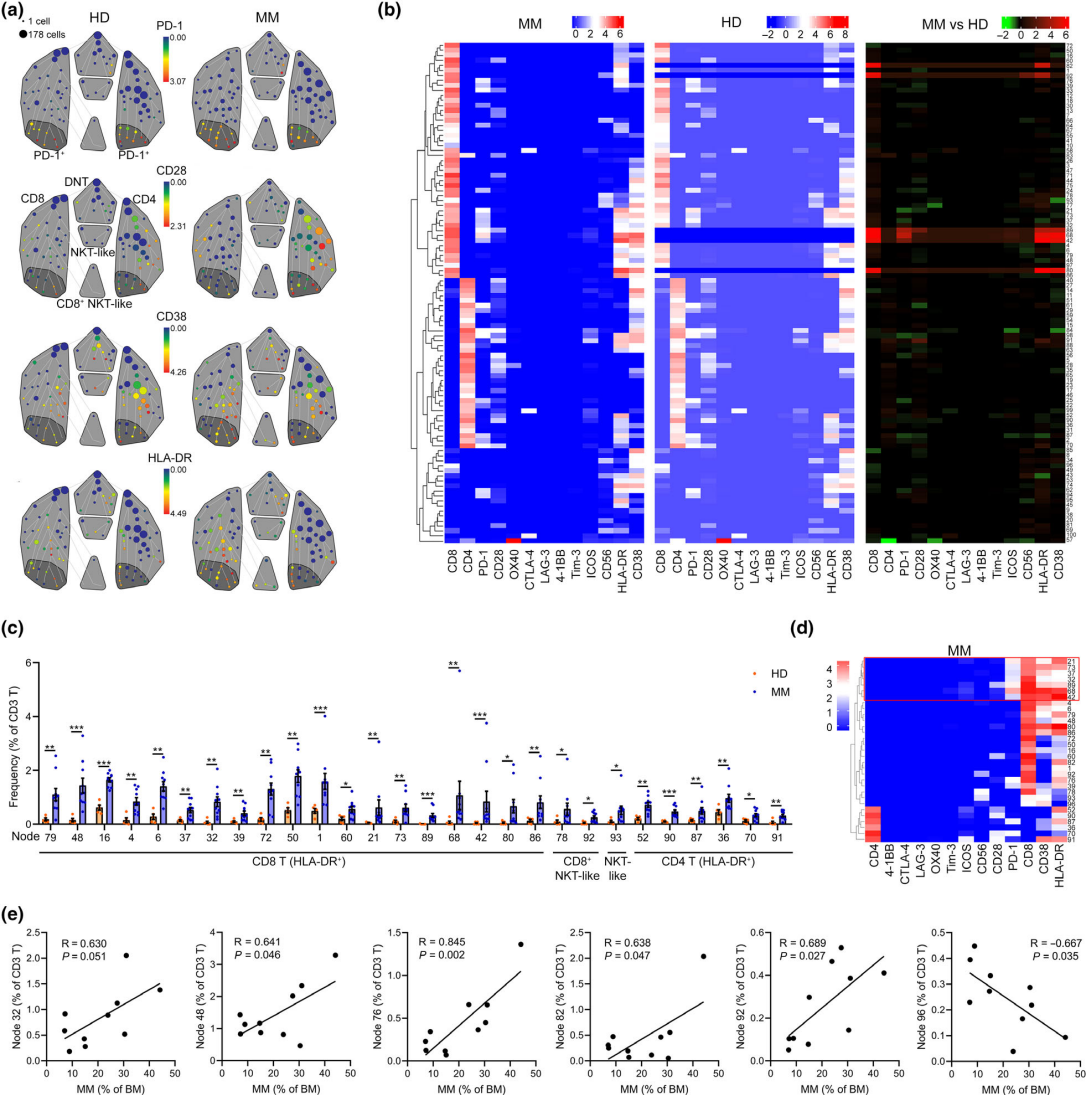
Identification of the immune checkpoint signature of T cell in MM patients. **(a)** A SPADE tree describing 100 small T‐cell clusters of one representative HD or MM patient coloured by the median expression of indicated markers. T‐cell subpopulations are gated with a grey colour, and PD‐1^+^ subsets are gated with a deep grey area. **(b)** Heatmaps showing the normalised median expression of indicated markers in 100 small T‐cell clusters of all MM patients and all HD and displaying the differences in markers' expression between T‐cell clusters of MM patients and HD (right panel). **(c)** Bar plots showing the significantly changed frequencies of T‐cell clusters (nodes) of HD and MM patients. **(d)** A heatmap showing the normalised median expression of indicated markers in significantly changed HLA‐R^+^ T‐cell clusters of MM patients. Red boxes indicate PD‐1^+^HLA‐DR^+^CD38^+^ CD8 T‐cell clusters. **(e)** Dot plots showing the Pearson correlation coefficients for relationships between the frequencies of MM cells and indicated T‐cell clusters. HD, *n* = 5; MM, *n* = 10. *P < 0.05, **P < 0.01 and ***P < 0.001.

As T cells are the primary anticancer contributor, we next systematically analysed the immune checkpoint phenotype of all possible exclusively and significantly changed T‐cell clusters. From the viSNE containing all CD3 T cells, we observed a huge heterogeneity of the T‐cell compartments, regarding the expression of immune modulatory proteins (Supplementary figure 7a). We next introduced spanning‐tree progression analysis of density‐normalised events (SPADE) analysis^40^ to divide all T cells into 100 minor clusters (nodes) containing cells with similar phenotypes. On the SPADE tree, we were able to characterise the immune checkpoint phenotype of each cluster and clearly observe the differences in these clusters in each individual (Figure 6a and Supplementary figure 7b). Using cytoClusterR, the heterogeneity of immune checkpoint receptor signatures across 100 T‐cell clusters from all 10 MM patients or five HD was obviously revealed on heatmaps (Figure 6b). Clusters 82, 92, 89, 68 and 42 were specifically presented in MM patients. In each cluster, different median expressions of immune checkpoint protein are summarised (Figure 6b). Among these 100 T‐cell clusters, the frequencies of 42 clusters in MM patients were significantly different from those in HD (Figure 6c and Supplementary figure 7c). Twenty‐eight clusters displayed an activated phenotype (HLA‐DR^+^) and were significantly increased in MM patients (Figure 6c), indicating that these T‐cell clusters may play pivotal roles in remodelling the MM BM immune microenvironment. Among these 28 clusters, eight CD8 T‐cell clusters, including clusters 37, 32, 39, 21, 73, 89, 68 and 42, were activated and PD‐1^+^, whereas all these clusters were deficient in CD28 expression, except cluster 89 (Figure 6d). In addition, MM burden was significantly correlated with the frequencies of clusters 32, 48, 76, 82, 92 and 96 in MM patients (Figure 6e), indicating that the changes in these T‐cell clusters are MM cell‐dependent.

### Immune checkpoint network in the MM BM microenvironment

**Figure 7:**
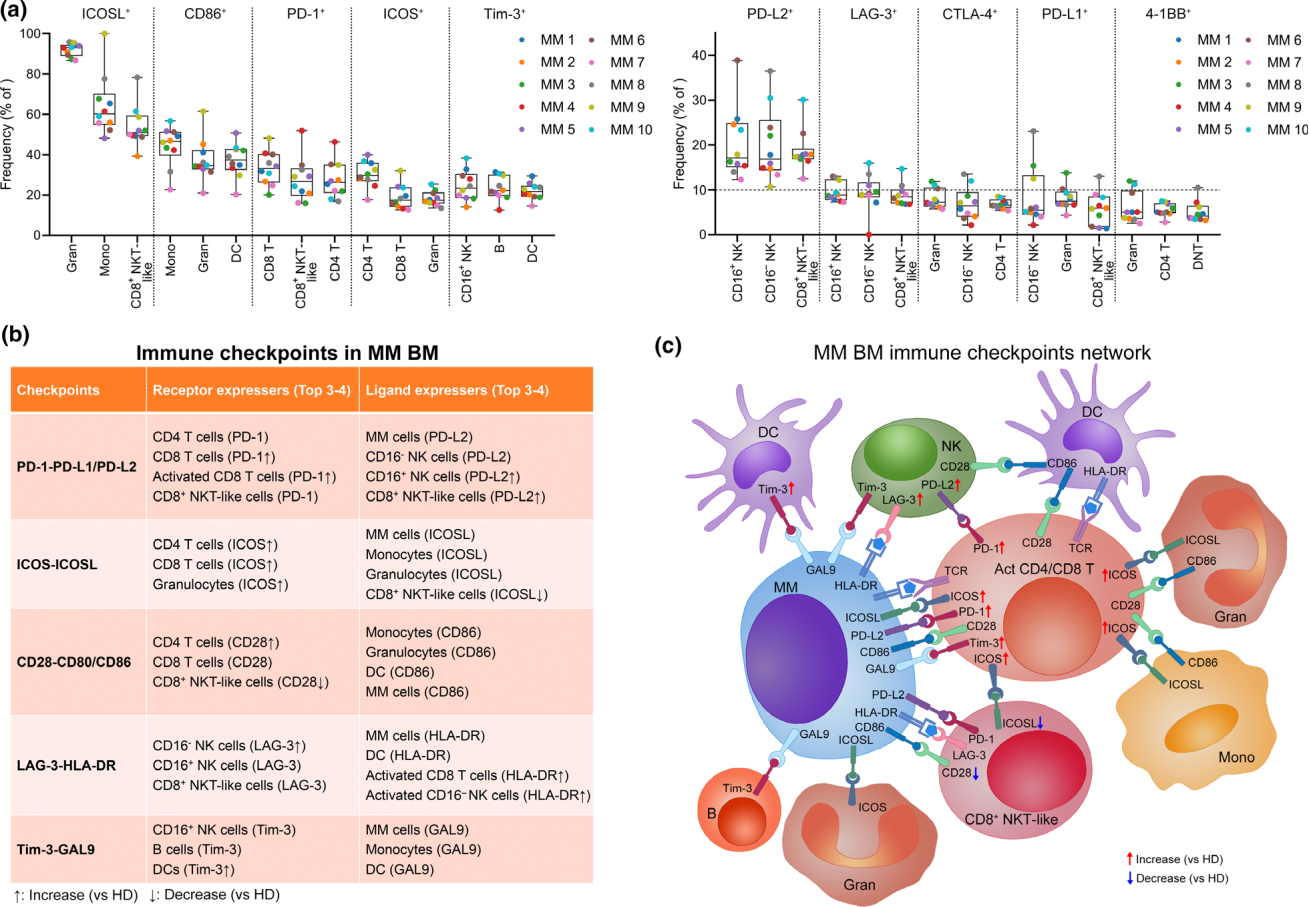
The immune checkpoint network in the MM BM microenvironment. **(a)** Dot plots showing the top 3 frequencies of indicated markers' positive cells in immune cell types. Dots are coloured by individual. **(b)** A table listing all the important checkpoints and their top 3 or 4 providers. **(c)** A schematic diagram showing the main provider cells of immune checkpoint ligands and receptors, and the network among them in the MM BM microenvironment. Act, activated. MM, *n* = 10.

We summarised the top 3 cell types providing immune checkpoint‐related receptors or ligands in MM patients (Figure 7a). Based on these main providers and the expression of immune checkpoint molecules in MM cells, a list and a network describing the interactions among MM and immune cells through immune checkpoints were established (Figure 7b and c). Considering the large heterogeneity among MM patients, we also built an immune checkpoint network for each MM patient (Supplementary figure 8).

## Discussion

The BM contains a complex environment and is filled with numerous kinds of immunoregulatory signal from both immune and non‐immune cells. In the MM BM microenvironment, non‐immune cells, such as stromal cells, regulate immunosuppression through cell‐to‐cell contact and release extracellular vesicles and thus favor immune evasion of MM cells.^41^ Immune checkpoints expressed on immune cells maintain the immune homeostasis, whereas MM cells enhance the suppression signal to escape from immune surveillance. Immune checkpoint blockade can break this malignant cell‐induced inhibitory communication and thus lead to the reinvigoration of anticancer immunity. Success of immune checkpoint therapies largely relies on the targets responsible for cancer‐induced immune suppression. To improve our understanding of the immune signature and immune checkpoint abnormalities in the MM BM microenvironment, we performed a high‐dimensional single‐cell analysis of the immune checkpoint molecules in healthy and MM BM samples. This high‐quality data set identifies an unambiguous immune checkpoint network in the MM immunologic milieu of these patients (Figure 7b and c) and establishes a powerful new level of insights into MM checkpoint immunotherapy.

Mass cytometry has been recently used to identify T‐cell heterogeneity and early alterations in resident T cells, and innate and myeloid cells in the BM of MM.^42^, ^43^ Kourelis *et al.* ^42^ have evaluated 33 immune markers, including five immune checkpoint molecules, in BM samples from dysproteinaemia patients, including MGUS, MM and AL amyloidosis, at diagnosis and after chemotherapy, and autologous stem cell transplant using mass cytometry. Similar to our results, they also found a very low level of CTLA‐4 in both CD4 and CD8 T cells and that PD‐1 is expressed by several T‐cell clusters, but not by all T cells. All identified BM cell types, except myeloid DC, express very low level of PD‐L1, further confirming the lack of PD‐1/PD‐L1 checkpoint signalling. The other recent study also analysed the BM T cells from 7 HD and 10 MM patients and the BM myeloid cells from 4 HD and 8 MM patients using mass cytometry.^43^ They discovered greater terminal effector differentiation in memory T cells and an increased PD‐L1 expression on myeloid cells from MM patients than healthy donors. However, detailed status of immune checkpoints, as well as the cell types providing checkpoint signals, has not been identified in these previous studies. Here, we devoted to systemically delineate the immune checkpoint signature of MM by measuring 10 pairs of immune checkpoint axes in freshly isolated BM samples from MM patients without treatment and our data would maximally reflect the real immune status of MM BM microenvironment.

Malignant cells offer a variety of immune checkpoint ligands to match receptors on immune cells and thus regulate anticancer immunity. With the successful application of PD‐1/PD‐L1 axis inhibitors in solid tumor immunotherapy, this blocking strategy has also become a focus of MM immunotherapy and plenty of clinical trials are conducted.^44^ However, single‐agent therapy with PD‐1 inhibitors fails to induce significant clinical responses in a phase 1b study,^16^ suggesting that PD‐1 blockade alone is insufficient to reinvigorate a clinically meaningful anti‐MM immunity. Discrepant results concerning PD‐L1 expression on MM cells have been reported.^45^ Several studies have confirmed the limited expression of PD‐L1 on MM cells^46^, ^47^, ^48^; in contrast, higher PD‐L1 has been also found in MM cells than plasma cells from HD.^5^, ^49^ Our comprehensive data revealed a low frequency (< 12%) of PD‐L1 expression in MM cells from all 10 MM patients. However, the expression of PD‐L2, another ligand for PD‐1, on MM cells was relatively higher than PD‐L1. Anyhow, ligands of PD‐1 were not widely expressed by MM cells, implicating the existence of other possible participants in inhibitory immunity. We validated here that several immune checkpoint ligands, including GAL9, ICOSL, HLA‐DR, CD86, PD‐L2 and 4‐1BBL, were more generally presented on MM cells and these ligands are able to largely influence the immune response through binding to their receptors on immune effector cells.

A significant positive correlation between MM burden and GAL9 expression, together with the high frequency of GAL9 expression on MM cells, emphasises the possible contribution of this ligand to the MM immune microenvironment. In addition, Tim‐3, a receptor of GAL9,^50^ was expressed by activated CD8 T, NKT‐like, DNT cells and DC in MM patients. Tim‐3‐GAL9 axis provides inhibitory immune signals to activated T cells,^51^ and immunotherapy targeting Tim‐3 and PD‐1 pathways enables the reversion of T‐cell exhaustion and restoration of antitumor immunity,^52^ thus suggesting a possible use of this strategy to reconstruct anti‐MM immunity.

ICOSL was also expressed by most of MM cells, and its receptor ICOS was increasingly detected in 20–40% of CD4 or CD8 T cells of MM patients. Being in line with this mechanism, a higher percentage of ICOS^+^ cells in follicular helper T cells has been found in MM patients than healthy controls.^53^ The ICOS/ICOSL signal can mediate helper T‐cell immunity and regulate effector T‐cell differentiation.^54^ *In vitro*, ICOS/ICOSL blockade significantly reduced the generation of MM cell‐induced inhibitory CD4^+^ Treg cells,^55^, ^56^ and lenalidomide, a clinically approved anti‐MM immunomodulatory drug, could inhibit ICOSL expression in MM cells^57^ and enhance PD‐1/PD‐L1 blockade‐induced anticancer immunity in MM patients.^58^ These evidences, together with our results, underline ICOS/ICOSL blockade as a possible enhancer for anti‐MM immunotherapeutic strategies.

T and NK cells are at the forefront of anticancer immune responses, and quantitative and functional abnormalities in these cells' subsets have been well identified in the MM BM microenvironment.^2^, ^59^, ^60^ The discovery of significant increases in CD4 T, CD8 T, CD16^+^ NK and CD8^+^ NKT‐like cells in MM BM compared with HD BM confirms an abnormal immune cell composition induced by MM cells. Remarkably, these increased T or NK cells are activated in the MM samples, but with a suppressive phenotype as several inhibitory receptors, such as PD‐1 and Tim‐3, were increased. Because of the fact that CTLA‐4, 4‐1BB and LAG‐3 were expressed only by very few CD8 T cells, targeting those checkpoints might be less effective. Deep analysis of T‐cell profiling identified several specifically activated CD8 T‐cell clusters highly expressing PD‐1 in MM patients, whereas most of them are deficient in CD28 expression, a critical T‐cell costimulatory receptor that binds to B7 molecules, including CD80 and CD86.^61^ The failure of PD‐1 inhibitors in MM immunotherapy may result from the deficiency of CD28 in activated CD8^+^ T cells as substantial evidences have demonstrated that successful reinvigoration of exhausted CD8^+^ T cells by PD‐1/PD‐L1 blockade is dependent on CD28.^62^, ^63^ Most likely, once CD28 signalling is restored in these increased numbers of activated CD8^+^ T cells, strong anti‐MM immunity will be achieved for controlling MM growth.

New targets or strategies are needed to increase the success of immune checkpoint‐based immunotherapy for MM. By fine‐grained analysis of the immune cells in the MM BM microenvironment, this study provides a detailed atlas of the infiltrating immune cells in MM, identifies immune checkpoints change that are unique to the MM immunologic milieu, and reveals distinct immune subsets that may be responsible for anti‐MM immunosuppression. These data will be a valuable resource for future research to explore more efficient immunotherapy strategies tailored to restore anti‐MM immunity through inhibition of immune checkpoints. The large individual heterogeneity in immune checkpoint networks among MM patients also emphasises the necessity of personalised strategies for a successful MM immunotherapy. Our findings demonstrating several potential immune checkpoint targets warrant further functional investigation into developing novel strategies for MM immunotherapy. In addition, non‐immune cell components, such as stromal cells and extracellular vesicles, which also play an important role in regulating immunosuppression in the MM BM, also need to be taken into account in discovering novel targets for MM treatment in future.

## Methods

### Human specimens

Multiple myeloma BM samples were collected from MM patients undergoing BM biopsy for diagnosis, and healthy BM samples were obtained from donors undergoing BM biopsy for BM donation. Informed consents in accordance with the Declaration of Helsinki were obtained from all participants. All participants were recruited at the Third Affiliated Hospital of Sun Yat‐sen University. All protocols were reviewed and approved by the Hospital Ethics Committee. The clinical characteristics of all participants are listed in Supplementary table 1.

### Sample processing

Bone marrow samples were collected into sodium heparin tubes. To maximally maintain the immune profile, freshly isolated BM cells were directly fixed using an optimised and well‐established fixing method with minimal effects on target epitope.^64^ About 1–2 mL of BM samples was fixed with Fix I Buffer (Fluidigm, South San Francisco, CA, USA) for 10 min at RT, and red blood lysis buffer was used to fully remove the erythrocytes. Cells were then resuspended in cell staining buffer (CSB) and Dulbecco's phosphate‐buffered saline, supplemented with 0.5% bovine serum albumin and 0.02% sodium azide, containing 10% dimethyl sulphoxide, and stored at −80°C until cell staining was performed.

### Barcoding

To eliminate sample‐specific staining variation, all samples were barcoded first and then stained, processed and acquired as one multiplexed sample. A total of 0.5 × 10^6^ fixed cells from each samples were washed thrice with CSB and washed twice with 1× Barcode Perm Buffer (Fluidigm). These samples were then barcoded using a 20‐Plex Pd Barcoding Kit (Fluidigm). Each sample was washed thrice with CSB after incubation with different barcodes for 30 min at RT, and all samples were combined together into one tube for antibody staining.

### Antibody staining

Combined samples were washed once with CSB and incubated with Human Fc Receptor Binding Inhibitor Antibody (Thermo Fisher, Waltham, MA, USA) for 10 min at RT to lower non‐specific binding. Anti‐human ICOSL‐biotin (BioLegend, San Diego, CA, USA) was added to the samples for incubation for another 30 min at RT. These cells were washed twice with CSB and stained with 29 metal isotope‐tagged antibodies and 1 metal‐labelled antibody against biotin (Supplementary table 2) for 30 min at RT. These stained cells were washed thrice with CSB and incubated with 1 mL Fix & Perm Buffer (Fluidigm) containing 125 nm Intercalator‐Ir (Fluidigm) overnight at 4°C.

### CyTOF data acquisition

Samples were washed twice with CSB and twice with ultrapure water. Immediately prior to data acquisition, the sample was resuspended in ultrapure water containing 15% EQ Four Element Calibration Beads (Fluidigm) and filtered through a 38‐μm cell strainer. The sample was acquired on a Helios mass cytometer (Fluidigm) at an acquisition rate of < 500 events/s. Bead‐based normalisation and debarcoding were completed using CyTOF software 6.7 (Fluidigm).

### Data analysis

Individual debarcoded files were uploaded to an online single‐cell analyser, Cytobank (Beckman Coulter, Brea, CA, USA).^65^ Beads, debris and doublets were excluded from the events, and the single‐cell data were subsequently used for high‐dimensional analyses. Contour plots, viSNE, SPADE and heatmaps were implemented using Cytobank. The frequency of positive cells in each gated population was determined using FlowJo (FlowJo LLC, Ashland, OR, USA). Bar plot, violin plot and heatmap of correlationship were generated using the GraphPad Prism software (GraphPad, San Diego, CA, USA). The comparison of HD and MM SPADE node was implemented using the cytoClustR R package developed in Kordasti Lab from King's College London. SPSS 20.0 software (IBM, Armonk, NY, USA) was used for the Pearson correlation analyses.

### Statistical analysis

The Mann–Whitney *U*‐test was used to determine the statistical significance between the two groups. A paired *t*‐test was performed on the frequencies of different cell subsets in individuals. Error bars represent mean ± standard error of mean (sem). A *P‐*value < 0.05 was considered as statistically significant.

## Conflict of interest

The authors declare no conflict of interest.

## Authors' contribution

JW and JL conceived the idea and supervised the experiments. YZ obtained the informed consents and collected samples. JW, CT and HZ implemented the experiments. JW analysed the data and wrote the manuscript. KV, EM and JL provided critical suggestions and revised the manuscript. All authors read and approved the final manuscript.

## Data Availability

Mass cytometry data that support the findings of this study are deposited in the FlowRepository database (No. FR‐FCM‐Z29D).

## References

[1] Wang J , Hendrix A , Hernot S *et al* Bone marrow stromal cell‐derived exosomes as communicators in drug resistance in multiple myeloma cells. Blood 2014; 124: 555–566. 24928860 10.1182/blood-2014-03-562439

[2] Dosani T , Carlsten M , Maric I , Landgren O . The cellular immune system in myelomagenesis: NK cells and T cells in the development of myeloma and their uses in immunotherapies. Blood Cancer J 2015; 5: e306. 26140429 10.1038/bcj.2015.49 PMC4526775

[3] Ghobrial I , Cruz CH , Garfall A *et al* Immunotherapy in multiple myeloma: accelerating on the path to the patient. Clin Lymphoma Myeloma Leuk 2019; 19: 332–344. 31023594 10.1016/j.clml.2019.02.004

[4] Ramachandran IR , Martner A , Pisklakova A *et al* Myeloid‐derived suppressor cells regulate growth of multiple myeloma by inhibiting T cells in bone marrow. J Immunol 2013; 190: 3815–3823. 23460744 10.4049/jimmunol.1203373 PMC3608837

[5] Liu J , Hamrouni A , Wolowiec D *et al* Plasma cells from multiple myeloma patients express B7–H1 (PD‐L1) and increase expression after stimulation with IFN‐γ and TLR ligands via a MyD88‐, TRAF6‐, and MEK‐dependent pathway. Blood 2007; 110: 296–304. 17363736 10.1182/blood-2006-10-051482

[6] Lucas F , Pennell M , Huang Y *et al* T cell transcriptional profiling and immunophenotyping uncover LAG3 as a potential significant target of immune modulation in multiple myeloma. Biol Blood Marrow Transplant 2020; 26: 7–15. 31445183 10.1016/j.bbmt.2019.08.009 PMC6952061

[7] Wang J , De Veirman K , Faict S *et al* Multiple myeloma exosomes establish a favourable bone marrow microenvironment with enhanced angiogenesis and immunosuppression. J Pathol 2016; 239: 162–173. 26956697 10.1002/path.4712

[8] Jelinek T , Mihalyova J , Kascak M , Duras J , Hajek R . PD‐1/PD‐L1 inhibitors in haematological malignancies: update 2017. Immunology 2017; 152: 357–371. 28685821 10.1111/imm.12788 PMC5629439

[9] Minn AJ , Wherry EJ . Combination cancer therapies with immune checkpoint blockade: convergence on interferon signaling. Cell 2016; 165: 272–275. 27058661 10.1016/j.cell.2016.03.031

[10] Pitt JM , Vetizou M , Daillere R *et al* Resistance mechanisms to immune‐checkpoint blockade in cancer: tumor‐intrinsic and ‐extrinsic factors. Immunity 2016; 44: 1255–1269. 27332730 10.1016/j.immuni.2016.06.001

[11] Syn NL , Teng MWL , Mok TSK , Soo RA . De‐novo and acquired resistance to immune checkpoint targeting. Lancet Oncol 2017; 18: e731–e741. 29208439 10.1016/S1470-2045(17)30607-1

[12] Topalian SL , Taube JM , Anders RA , Pardoll DM . Mechanism‐driven biomarkers to guide immune checkpoint blockade in cancer therapy. Nat Rev Cancer 2016; 16: 275–287. 27079802 10.1038/nrc.2016.36 PMC5381938

[13] Wilson RAM , Evans TRJ , Fraser AR , Nibbs RJB . Immune checkpoint inhibitors: new strategies to checkmate cancer. Clin Exp Immunol 2018; 191: 133–148. 29139554 10.1111/cei.13081 PMC5758374

[14] Brunner‐Weinzierl MC , Rudd CE . CTLA‐4 and PD‐1 control of T‐cell motility and migration: implications for tumor immunotherapy. Front Immunol 2018; 9: 2737. 30542345 10.3389/fimmu.2018.02737 PMC6277866

[15] Jenkins RW , Barbie DA , Flaherty KT . Mechanisms of resistance to immune checkpoint inhibitors. Br J Cancer 2018; 118: 9–16. 29319049 10.1038/bjc.2017.434 PMC5765236

[16] Suen H , Brown R , Yang S *et al* The failure of immune checkpoint blockade in multiple myeloma with PD‐1 inhibitors in a phase 1 study. Leukemia 2015; 29: 1621–1622. 25987102 10.1038/leu.2015.104

[17] Lesokhin AM , Ansell SM , Armand P *et al* Nivolumab in patients with relapsed or refractory hematologic malignancy: preliminary results of a phase Ib study. J Clin Oncol 2016; 34: 2698–2704. 27269947 10.1200/JCO.2015.65.9789 PMC5019749

[18] Pardoll DM . The blockade of immune checkpoints in cancer immunotherapy. Nat Rev Cancer 2012; 12: 252–264. 22437870 10.1038/nrc3239 PMC4856023

[19] Bendall SC , Simonds EF , Qiu P *et al* Single‐cell mass cytometry of differential immune and drug responses across a human hematopoietic continuum. Science 2011; 332: 687–696.21551058

[20] Bodenmiller B , Zunder ER , Finck R *et al* Multiplexed mass cytometry profiling of cellular states perturbed by small‐molecule regulators. Nat Biotechnol 2012; 30: 858–867. 22902532 10.1038/nbt.2317 PMC3627543

[21] Bendall SC , Davis KL , el Amir AD *et al* Single‐cell trajectory detection uncovers progression and regulatory coordination in human B cell development. Cell 2014; 157: 714–725. 24766814 10.1016/j.cell.2014.04.005 PMC4045247

[22] Porpiglia E , Samusik N , Ho ATV *et al* High‐resolution myogenic lineage mapping by single‐cell mass cytometry. Nat Cell Biol 2017; 19: 558–567. 28414312 10.1038/ncb3507 PMC5728993

[23] Lavin Y , Kobayashi S , Leader A *et al* Innate immune landscape in early lung adenocarcinoma by paired single‐cell analyses. Cell 2017; 169: 750–765.e17. 28475900 10.1016/j.cell.2017.04.014 PMC5737939

[24] Chevrier S , Levine JH , Zanotelli VRT *et al* An immune atlas of clear cell renal cell carcinoma. Cell 2017; 169: 736–749.e18. 28475899 10.1016/j.cell.2017.04.016 PMC5422211

[25] Wagner J , Rapsomaniki MA , Chevrier S *et al* A single‐cell atlas of the tumor and immune ecosystem of human breast cancer. Cell 2019; 177: 1330–1345.e18. 30982598 10.1016/j.cell.2019.03.005 PMC6526772

[26] Lin P , Owens R , Tricot G , Wilson CS . Flow cytometric immunophenotypic analysis of 306 cases of multiple myeloma. Am J Clin Pathol 2004; 121: 482–488. 15080299 10.1309/74R4-TB90-BUWH-27JX

[27] Reid S , Yang S , Brown R *et al* Characterisation and relevance of CD138‐negative plasma cells in plasma cell myeloma. Int J Lab Hematol 2010; 32: e190–e196. 20201998 10.1111/j.1751-553X.2010.01222.x

[28] el Amir AD , Davis KL , Tadmor MD *et al* viSNE enables visualization of high dimensional single‐cell data and reveals phenotypic heterogeneity of leukemia. Nat Biotechnol 2013; 31: 545–552. 23685480 10.1038/nbt.2594 PMC4076922

[29] Maecker HT , McCoy JP , Nussenblatt R . Standardizing immunophenotyping for the human immunology project. Nat Rev Immunol 2012; 12: 191–200. 22343568 10.1038/nri3158 PMC3409649

[30] Chan WK , Rujkijyanont P , Neale G *et al* Multiplex and genome‐wide analyses reveal distinctive properties of KIR^+^ and CD56^+^ T cells in human blood. J Immunol 2013; 191: 1625–1636. 23858032 10.4049/jimmunol.1300111 PMC4275795

[31] Campbell JJ , Qin S , Unutmaz D *et al* Unique subpopulations of CD56^+^ NK and NK‐T peripheral blood lymphocytes identified by chemokine receptor expression repertoire. J Immunol 2001; 166: 6477–6482. 11359797 10.4049/jimmunol.166.11.6477

[32] Krijgsman D , Hokland M , Kuppen PJK . The role of natural killer T cells in cancer‐a phenotypical and functional approach. Front Immunol 2018; 9: 367. 29535734 10.3389/fimmu.2018.00367 PMC5835336

[33] Tiwary S , Berzofsky JA , Terabe M . Altered lipid tumor environment and its potential effects on NKT cell function in tumor immunity. Front Immunol 2019; 10: 2187. 31620124 10.3389/fimmu.2019.02187 PMC6759687

[34] Arruvito L , Payaslian F , Baz P *et al* Identification and clinical relevance of naturally occurring human CD8^+^HLA‐DR^+^ regulatory T cells. J Immunol 2014; 193: 4469–4476. 25261474 10.4049/jimmunol.1401490

[35] Evans JH , Horowitz A , Mehrabi M *et al* A distinct subset of human NK cells expressing HLA‐DR expand in response to IL‐2 and can aid immune responses to BCG. Eur J Immunol 2011; 41: 1924–1933. 21491418 10.1002/eji.201041180 PMC3549558

[36] Christopoulos P , Pfeifer D , Bartholome K *et al* Definition and characterization of the systemic T‐cell dysregulation in untreated indolent B‐cell lymphoma and very early CLL. Blood 2011; 117: 3836–3846. 21270444 10.1182/blood-2010-07-299321

[37] McElroy AK , Akondy RS , Davis CW *et al* Human Ebola virus infection results in substantial immune activation. Proc Natl Acad Sci USA 2015; 112: 4719–4724. 25775592 10.1073/pnas.1502619112 PMC4403189

[38] Simoni Y , Becht E , Fehlings M *et al* Bystander CD8^+^ T cells are abundant and phenotypically distinct in human tumour infiltrates. Nature 2018; 557: 575–579. 29769722 10.1038/s41586-018-0130-2

[39] Duhen T , Duhen R , Montler R *et al* Co‐expression of CD39 and CD103 identifies tumor‐reactive CD8 T cells in human solid tumors. Nat Commun 2018; 9: 2724. 30006565 10.1038/s41467-018-05072-0 PMC6045647

[40] Qiu P , Simonds EF , Bendall SC *et al* Extracting a cellular hierarchy from high‐dimensional cytometry data with SPADE. Nat Biotechnol 2011; 29: 886–891. 21964415 10.1038/nbt.1991 PMC3196363

[41] Wang J , De Veirman K , De Beule N *et al* The bone marrow microenvironment enhances multiple myeloma progression by exosome‐mediated activation of myeloid‐derived suppressor cells. Oncotarget 2015; 6: 43992–44004. 26556857 10.18632/oncotarget.6083 PMC4791281

[42] Kourelis TV , Villasboas JC , Jessen E *et al* Mass cytometry dissects T cell heterogeneity in the immune tumor microenvironment of common dysproteinemias at diagnosis and after first line therapies. Blood Cancer J 2019; 9: 72. 31462637 10.1038/s41408-019-0234-4 PMC6713712

[43] Bailur JK , McCachren SS , Doxie DB *et al* Early alterations in stem‐like/resident T cells, innate and myeloid cells in the bone marrow in preneoplastic gammopathy. JCI Insight 2019; 5: 127807. 31013254 10.1172/jci.insight.127807 PMC6629164

[44] Lesokhin AM , Bal S , Badros AZ . Lessons learned from checkpoint blockade targeting PD‐1 in multiple myeloma. Cancer Immunol Res 2019; 7: 1224–1229. 31371317 10.1158/2326-6066.CIR-19-0148 PMC6891823

[45] Jelinek T , Paiva B , Hajek R . Update on PD‐1/PD‐L1 inhibitors in multiple myeloma. Front Immunol 2018; 9: 2431. 30505301 10.3389/fimmu.2018.02431 PMC6250817

[46] Paiva B , Azpilikueta A , Puig N *et al* PD‐L1/PD‐1 presence in the tumor microenvironment and activity of PD‐1 blockade in multiple myeloma. Leukemia 2015; 29: 2110–2113. 25778100 10.1038/leu.2015.79

[47] Kelly KR , Espitia CM , Zhao W *et al* Oncolytic reovirus sensitizes multiple myeloma cells to anti‐PD‐L1 therapy. Leukemia 2018; 32: 230–233. 28832023 10.1038/leu.2017.272 PMC5844271

[48] Favreau M , Venken K , Faict S *et al* Both mucosal‐associated invariant and natural killer T‐cell deficiency in multiple myeloma can be countered by PD‐1 inhibition. Haematologica 2017; 102: e266–e270. 28385777 10.3324/haematol.2017.163758 PMC5566052

[49] Tamura H , Ishibashi M , Yamashita T *et al* Marrow stromal cells induce B7–H1 expression on myeloma cells, generating aggressive characteristics in multiple myeloma. Leukemia 2013; 27: 464–472. 22828443 10.1038/leu.2012.213

[50] Zhu C , Anderson AC , Schubart A *et al* The Tim‐3 ligand galectin‐9 negatively regulates T helper type 1 immunity. Nat Immunol 2005; 6: 1245–1252. 16286920 10.1038/ni1271

[51] Perez‐Gracia JL , Labiano S , Rodriguez‐Ruiz ME , Sanmamed MF , Melero I . Orchestrating immune check‐point blockade for cancer immunotherapy in combinations. Curr Opin Immunol 2014; 27: 89–97. 24485523 10.1016/j.coi.2014.01.002

[52] Sakuishi K , Apetoh L , Sullivan JM *et al* Targeting Tim‐3 and PD‐1 pathways to reverse T cell exhaustion and restore anti‐tumor immunity. J Exp Med 2010; 207: 2187–2194. 20819927 10.1084/jem.20100643 PMC2947065

[53] Zhou DM , Xu YX , Zhang LY *et al* The role of follicular T helper cells in patients with malignant lymphoid disease. Hematology 2017; 22: 412–418. 28281408 10.1080/10245332.2017.1300623

[54] Wikenheiser DJ , Stumhofer JS . ICOS co‐stimulation: friend or foe? Front Immunol 2016; 7: 304. 27559335 10.3389/fimmu.2016.00304 PMC4979228

[55] Feyler S , Scott GB , Parrish C *et al* Tumour cell generation of inducible regulatory T‐cells in multiple myeloma is contact‐dependent and antigen‐presenting cell‐independent. PLoS One 2012; 7: e35981. 22666318 10.1371/journal.pone.0035981 PMC3362588

[56] Raja KRM , Hajek R . Contribution of regulatory T cells to immunosuppression and disease progression in multiple myeloma patients. Oncoimmunology 2013; 2: e25619. 24327932 10.4161/onci.25619 PMC3850022

[57] Scott GB , Carter C , Parrish C , Wood PM , Cook G . Downregulation of myeloma‐induced ICOS‐L and regulatory T cell generation by lenalidomide and dexamethasone therapy. Cell Immunol 2015; 297: 1–9. 26051632 10.1016/j.cellimm.2015.05.002

[58] Gorgun G , Samur MK , Cowens KB *et al* Lenalidomide enhances immune checkpoint blockade‐induced immune response in multiple myeloma. Clin Cancer Res 2015; 21: 4607–4618. 25979485 10.1158/1078-0432.CCR-15-0200 PMC4609232

[59] Perez‐Andres M , Almeida J , Martin‐Ayuso M *et al* Characterization of bone marrow T cells in monoclonal gammopathy of undetermined significance, multiple myeloma, and plasma cell leukemia demonstrates increased infiltration by cytotoxic/Th1 T cells demonstrating a squed TCR‐Vβ repertoire. Cancer 2006; 106: 1296–1305. 16475149 10.1002/cncr.21746

[60] Tamura H . Immunopathogenesis and immunotherapy of multiple myeloma. Int J Hematol 2018; 107: 278–285. 29368256 10.1007/s12185-018-2405-7

[61] Esensten JH , Helou YA , Chopra G , Weiss A , Bluestone JA . CD28 costimulation: from mechanism to therapy. Immunity 2016; 44: 973–988. 27192564 10.1016/j.immuni.2016.04.020 PMC4932896

[62] Kamphorst AO , Wieland A , Nasti T *et al* Rescue of exhausted CD8 T cells by PD‐1‐targeted therapies is CD28‐dependent. Science 2017; 355: 1423–1427. 28280249 10.1126/science.aaf0683 PMC5595217

[63] Hui EF , Cheung J , Zhu J *et al* T cell costimulatory receptor CD28 is a primary target for PD‐1‐mediated inhibition. Science 2017; 355: 1428–1433. 28280247 10.1126/science.aaf1292 PMC6286077

[64] Krutzik PO , Clutter MR , Nolan GP . Coordinate analysis of murine immune cell surface markers and intracellular phosphoproteins by flow cytometry. J Immunol 2005; 175: 2357–2365. 16081806 10.4049/jimmunol.175.4.2357

[65] Kotecha N , Krutzik PO , Irish JM . Web‐based analysis and publication of flow cytometry experiments. Curr Protoc Cytom 2010; Chapter 10: Unit10.17. 10.1002/0471142956.cy1017s53 PMC4208272 20578106

