# Supplementary material for: Identification of the immune checkpoint signature of multiple myeloma using mass cytometry‐based single‐cell analysis
Source: Clin Transl Immunology. 2020 Apr 29;9(5):e1132. doi: 10.1002/cti2.1132 (PMC7190397; doi:10.1002/cti2.1132)
Supplement: Supplementary file 1 [file CTI2-9-e1132-s001.pdf]

Supplementary material

Supplementary method

Antibody staining of CD39, CD103 and TCR Va24Ja18 in T cells

HD and MM cells were barcoded first and combined together into one tube for antibody staining after separately washing. Combined samples were washed once with CSB and incubated with Human Fc Receptor Binding Inhibitor Antibody (Thermo Fisher) for 10 min at RT to lower non-specific binding. Anti-human CD39-PE, anti-human CD103-APC and anti-human TCR Va24Ja18-FITC (Biolegend) were added to the samples for incubation for another 30 min at RT. These cells were washed twice with CSB and stained with 7 metal isotope-tagged antibodies and 3 metal-labeled antibodies against PE, APC, or FITC (Supplementary table 3) for 30 min at RT. These stained cells were washed 3 times with CSB and incubated with 1mL Fix & Perm Buffer (Fluidigm) containing 125 nM Intercalator-Ir (Fluidigm) overnight at 4 ^{o}C.

Cytoscape analysis

Cell subsets containing more than 20% indicated immune checkpoint protein-expressing cells in individual were included for Cytoscape analysis. Cytoscape 3.7.1 was used for visualizing complex networks of immune checkpoint among different cell populations.

Supplementary tables

Supplementary table 1. Clinical characteristics of healthy donor (HD) and patients

No.	Age	Sex	Disease	M-component type	Therapy
HD1	42	Female	N/A	N/A	N/A
HD2	59	Female	N/A	N/A	N/A
HD 3	55	Female	N/A	N/A	N/A
HD 4	37	Male	N/A	N/A	N/A
HD 5	50	Male	N/A	N/A	N/A
MM1	60	Male	MM	IgG κ	No*
MM2	51	Male	MM	IgG κ	No*
MM3	68	Male	MM	λ light chain	No*
MM4	59	Male	MM	IgG κ	No*
MM5	62	Female	MM	IgG λ	No*
MM6	51	Male	MM	IgG κ	No*
MM7	58	Male	MM	κ light chain	No*
MM8	70	Male	MM	IgG κ	No*
MM9	50	Female	MM	λ light chain	No*
MM10	57	Male	MM	IgG λ	No*

*No chemotherapy was received before sample collection. All these patients were newly diagnosed with MM. N/A, not applicable.

Supplementary table 2. Mass cytometry antibody panel 1

Label	Antigen	Ab clone	Dilution	Manufacturer
142Nd	CD19	HIB19	1:100	Fluidigm
143Nd	CD278 (ICOS)	C398.4A	1:100	Fluidigm
144Nd	CD11b (Mac-1)	ICRF44	1:100	Fluidigm
145Nd	CD138	DL-101	1:100	Fluidigm
147Sm	CD11c	Bu15	1:100	Fluidigm
148Nd	CD16	3G8	1:100	Fluidigm
150Nd	CD134 (OX40)	ACT35	1:100	Fluidigm
149Sm	CD56 (NCAM)	NCAM16.2	1:100	Fluidigm
151Eu	HLA-DR	G46-6	1:100	Fluidigm
152Sm	CD66b	80H3	1:100	Fluidigm
153Eu	TIM-3	F38-2E2	1:100	Fluidigm
154Sm	CD3	UCHT1	1:100	Fluidigm
155Gd	CD279 (PD-1)	EH12.2H7	1:100	Fluidigm
156Gd	CD86	IT2.2	1:100	Fluidigm
158Gd	CD137L (4-1BBL)	5F4	1:100	Fluidigm
159Tb	CD274 (PD-L1)	29E.2A3	1:100	Fluidigm

160Gd	CD28	CD28.2	1:100	Fluidigm
161Dy	CD152 (CTLA-4)	14D3	1:100	Fluidigm
162Dy	CD80 (B7-1)	2D10.4	1:100	Fluidigm
163Dy	Galectin-9	9M1-3	1:100	Fluidigm
165Ho	CD223 (LAG-3)	11C3C65	1:100	Fluidigm
167Er	CD38	HIT2	1:100	Fluidigm
168Er	CD8a	SK1	1:100	Fluidigm
169Tm	CD33	WM53	1:100	Fluidigm
172Yb	CD273 (PD-L2)	24F.10C12	1:100	Fluidigm
173Yb	CD137/4-1BB	4B4-1	1:100	Fluidigm
174Yb	CD4	SK3	1:100	Fluidigm
175Lu	CD14	M5E2	1:100	Fluidigm
89Y	CD45	HI30	1:100	Fluidigm
170Er	Biotin-CD275	1D4-C5	1:100	Fluidigm
Biotin	CD275 (ICOSL)	2D3	1:20	Biolegend

Supplementary table 3. Mass cytometry antibody panel 2

Label	Antigen	Ab clone	Dilution	Manufacturer
89Y	CD45	HI30	1:100	Fluidigm
145Nd	PE	PE001	1:100	Fluidigm
149Sm	CD56 (NCAM)	NCAM16.2	1:100	Fluidigm
151Eu	HLA-DR	G46-6	1:100	Fluidigm
154Sm	CD3	UCHT1	1:100	Fluidigm
160Gd	FITC	FIT-22	1:100	Fluidigm
167Er	CD38	HIT2	1:100	Fluidigm
168Er	CD8a	SK1	1:100	Fluidigm
174Yb	CD4	SK3	1:100	Fluidigm
176Yb	APC	APC003	1:100	Fluidigm
PE	CD39	A1	1:20	Biolegend
APC	CD103	Ber-ACT8	1:20	Biolegend
FITC	Vα24-Jα18	6B11	1:20	Biolegend

Supplementary figures

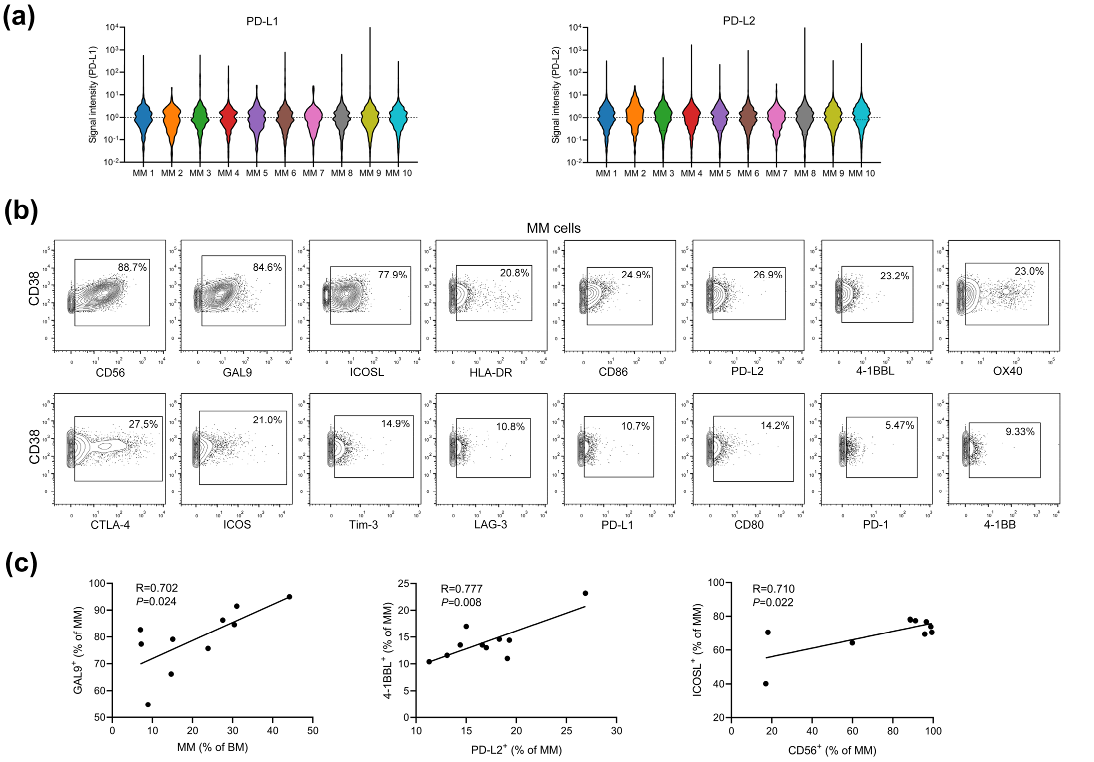

Supplementary figure 1. (a) Violin plot showing the signal intensity of PD-L1, and PD-L2 in MM cells of individual patients. **(b)** Contour plots showing the gating strategy and the expression of indicated checkpoint molecules in MM cells of one representative MM patient. **(c)** Dot plots showing Pearson correlation coefficients for relationships between the frequencies of indicated cell populations. MM, n=10.

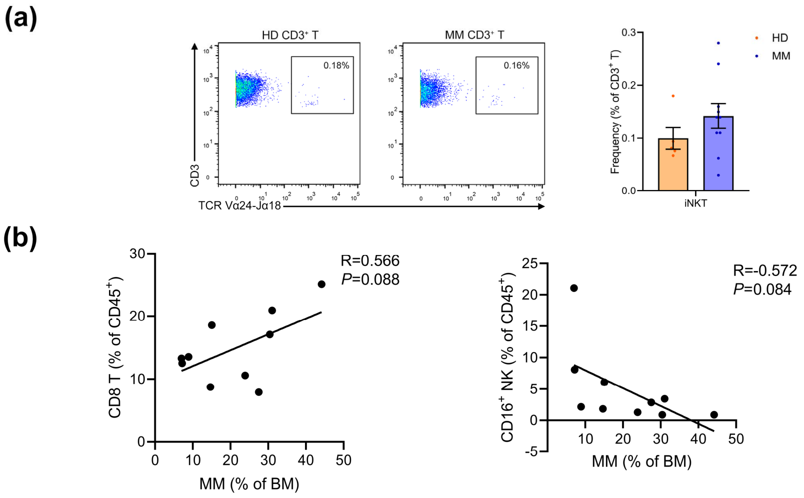

Supplementary figure 2. (a) Pseudocolor plots illustrating the iNKT cell gating strategy in the BM of one representative HD and one MM patient. Bar plots showing

iNKT cell percentages within BM T cells in HD and MM patients. **(b)** Dot plots showing Pearson correlation coefficients for relationships between the frequencies of indicated cell populations. HD, n=5; MM, n=10.

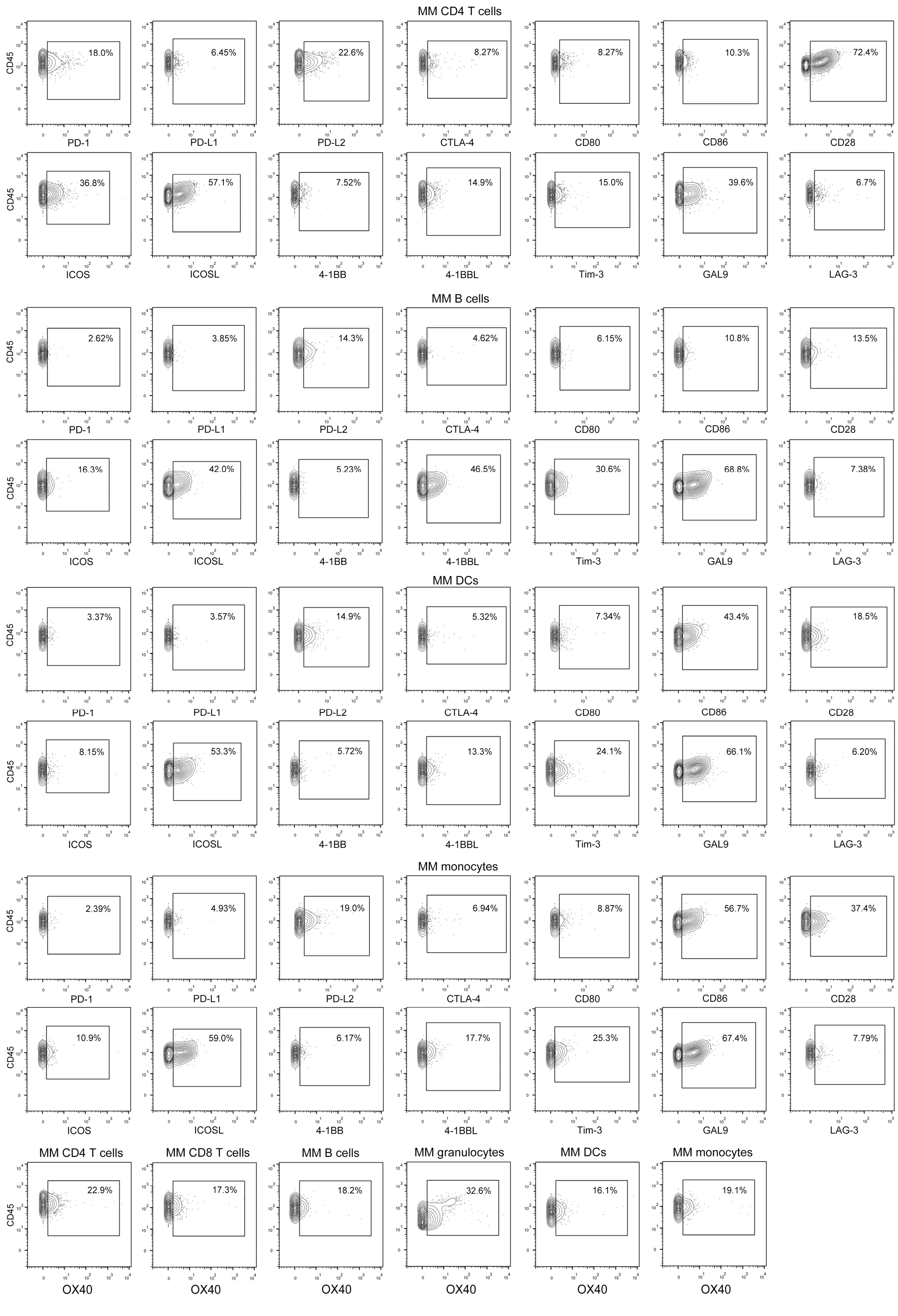

Supplementary figure 3. Contour plots showing the gating strategy and the expression of indicated checkpoint molecules in the indicated cell populations of one

representative MM patient.

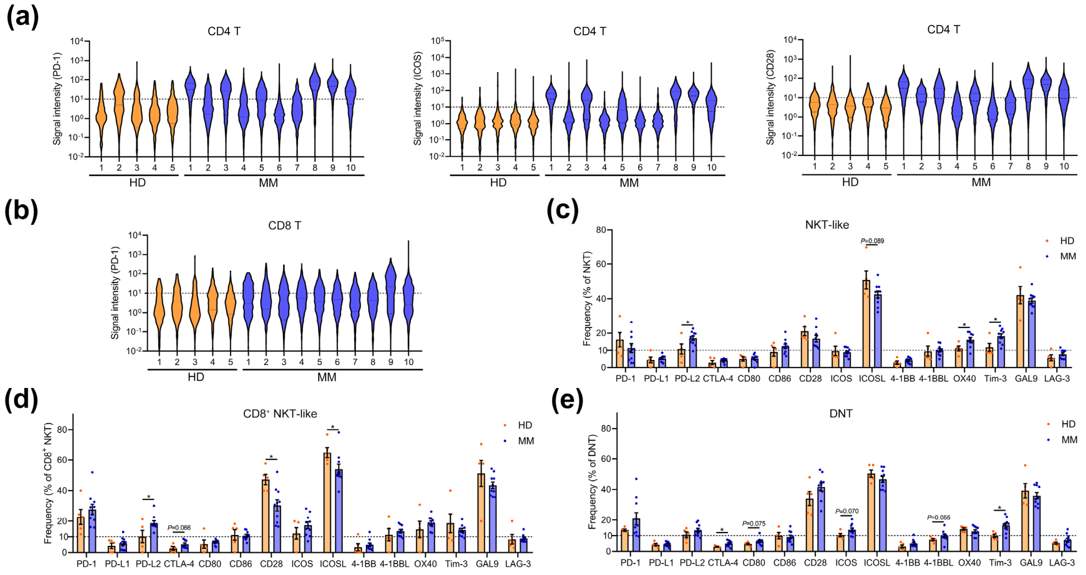

Supplementary figure 4. (a) Violin plot showing the signal intensity of PD-1, ICOS, and CD28 in CD4 T cells. (b) Violin plot showing the signal intensity of PD-1 in CD8 T cells. (c-e) Bar plots showing the frequencies of indicated markers' positive cells in BM **(c)** NKT-like, **(d)** $CD8^+$ NKT-like, and **(e)** DNT cells of HD and MM patients. HD, n=5; MM, n=10. * $P < 0.05$.

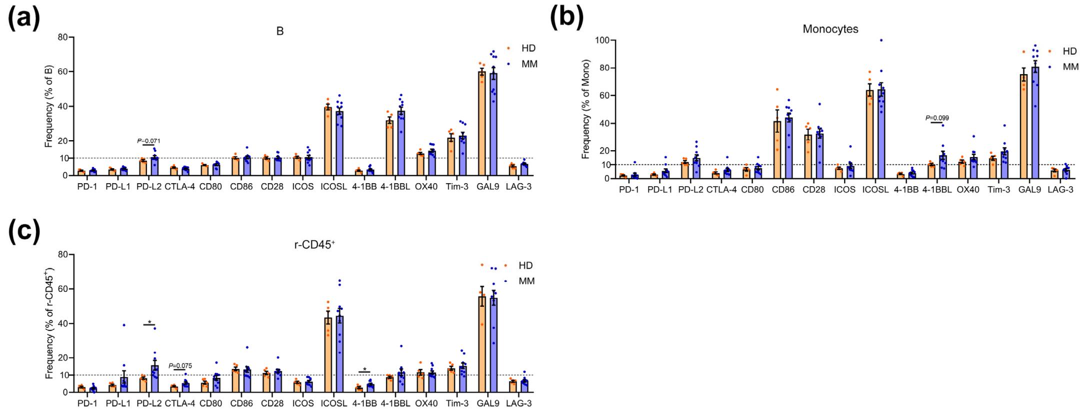

Supplementary figure 5. Bar plots showing the frequencies of indicated markers' positive cells in BM **(a)** B, **(b)** Monocytes, and **(c)** r-$CD45^+$ cells of HD and MM patients. HD, n=5; MM, n=10. * $P < 0.05$.

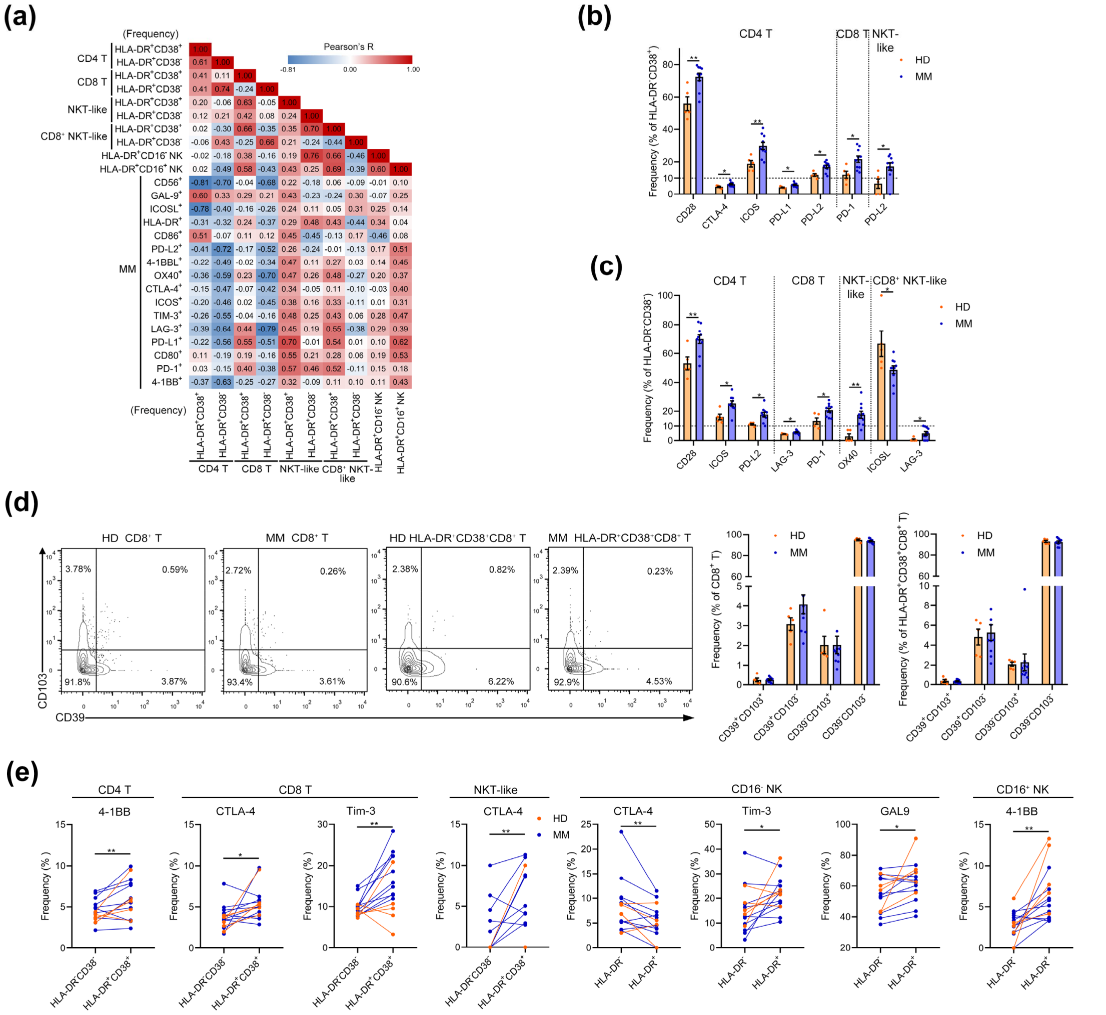

Supplementary figure 6. (a) Heatmap showing Pearson correlation coefficients for relationships between the frequencies of indicated cell subsets. **(b and c)** Bar plots showing the significantly changed median signal intensity of indicated markers in corresponding positive cells in **(b)** HLA-DR$^-$CD38$^+$ and **(c)** HLA-DR$^-$CD38$^-$ T cell subsets of HD and MM patients. **(d)** Contour Plots illustrating the gating strategy and the expression of CD39 and CD103 cells in CD8$^+$ and HLA-DR$^+$CD38$^+$CD8$^+$ T cells of one representative healthy donor (HD) or MM patient. Bar plots showing the frequencies of indicated cell clusters in BM T subsets cells of HD and MM patients. **(e)** Dot plots showing the showing the significantly changed frequencies of indicated markers' positive cells in HLA-DR$^-$CD38$^-$ and HLA-DR$^+$CD38$^+$ T cell subsets of individual and displaying the significantly changed frequencies of indicated markers' positive cells in HLA-DR$^-$ and HLA-DR$^+$ NK cell subsets of individual. HD, n=5; MM, n=10. * $P < 0.05$, ** $P < 0.01$.

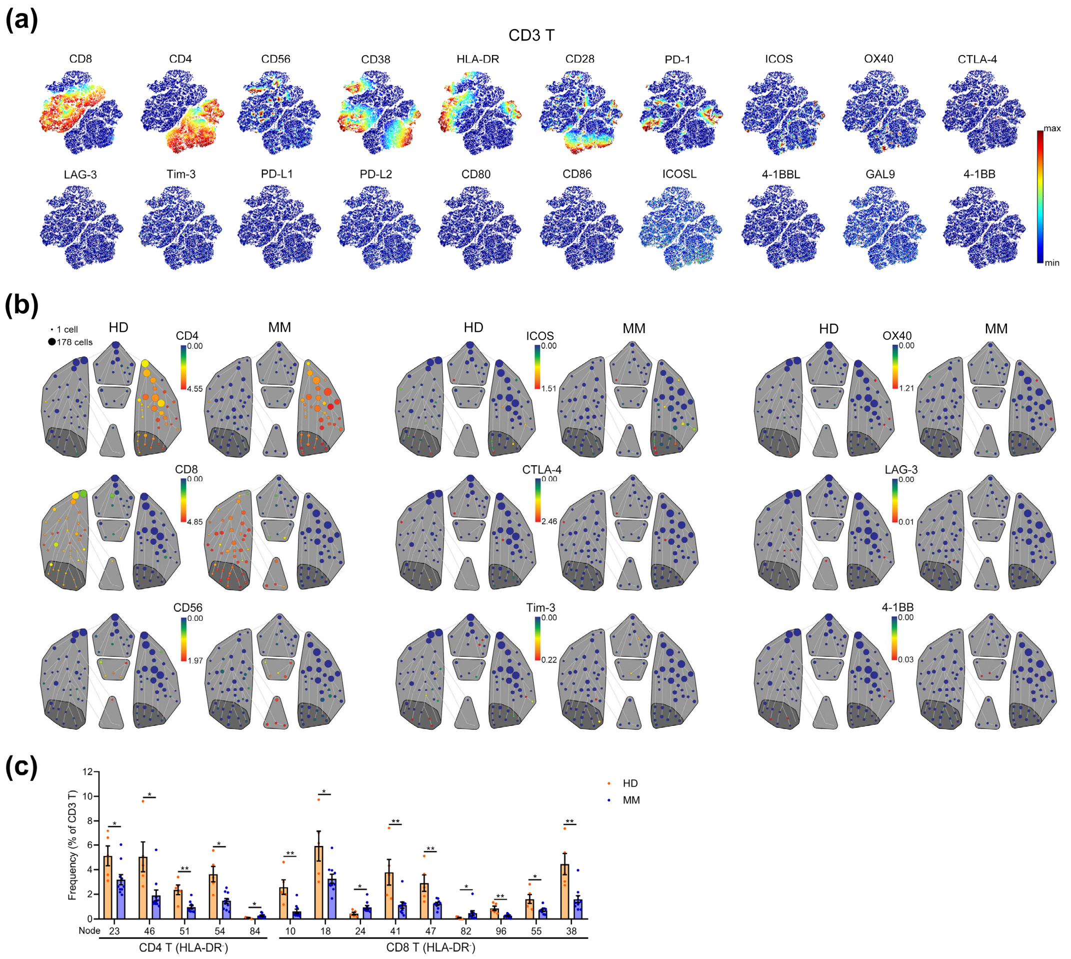

Supplementary figure 7. (a) viSNE map displaying all HD and MM CD3 T cells colored by normalized expression of 20 makers. **(b)** SPADE tree describing 100 minor T cell clusters of representative HD and MM patient colored by the median expression of indicated markers. T cell subpopulations are gated with a grey color, and PD-1^{+} subsets are gated with a deep grey area. **(c)** Bar plots showing the significantly changed frequencies of HLA-DR^{-} T cell clusters (nodes) of HD and MM patients. HD, n=5; MM, n=10. * $P < 0.05$, ** $P < 0.01$.

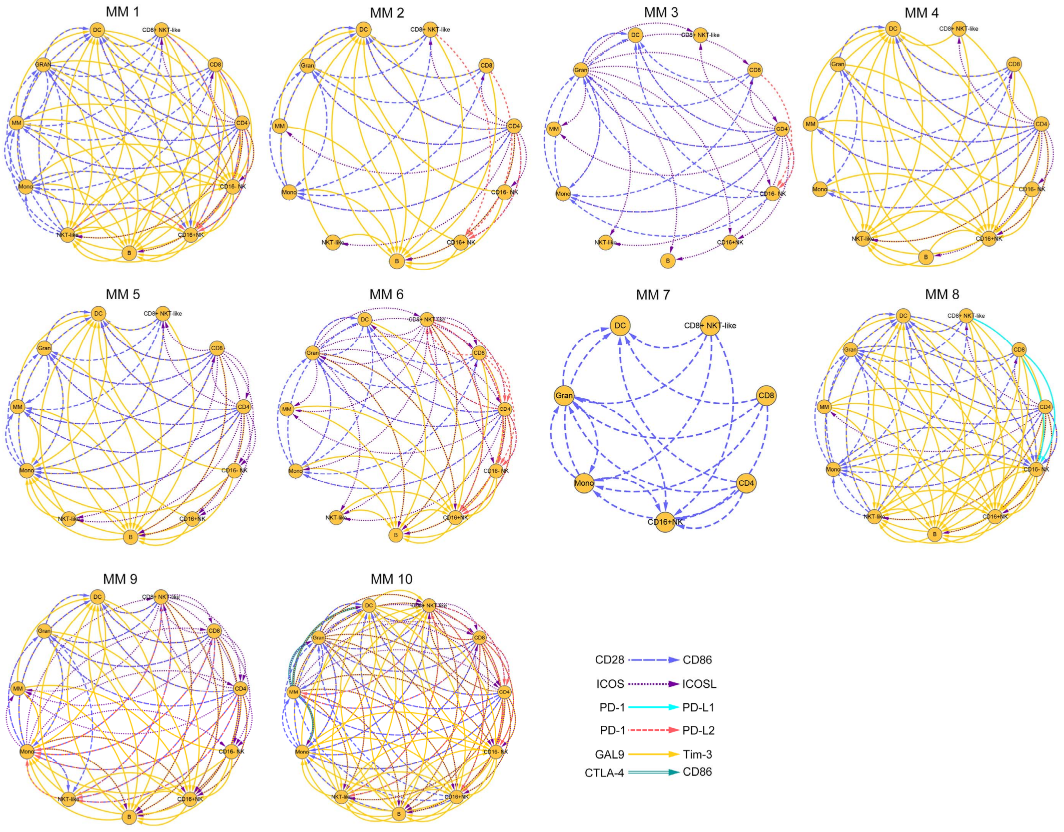

Supplementary figure 8. Immune checkpoint networks in individual MM patients. Only cell subsets containing more than 20% indicated immune checkpoint protein-expressing cells in individual were included for network establishment.
